# Neutrophils close the loop: self-sustaining splenic myelopoiesis in cancer

**DOI:** 10.1172/JCI213855

**Published:** 2026-11-16

**Authors:** Hongpeng Jia

**Affiliations:** Division of Pediatric Surgery, Department of Surgery, Johns Hopkins University School of Medicine, Baltimore, Maryland, USA.

## Abstract

Cancer-associated extramedullary hematopoiesis is increasingly recognized as a systemic mechanism of immune evasion. Although the spleen is an important site of tumor-induced myelopoiesis, how this process is locally maintained remains incompletely understood. In this issue of the *JCI*, Zhu and colleagues identify a self-reinforcing circuit between tumor-associated neutrophils and splenic hematopoietic stem and progenitor cells (HSPCs). They show that neutrophil-derived, NOX2-dependent ROS activated forkhead box O1 (FOXO1) in neighboring HSPCs, inducing dynamin-related protein 1 dependent (DRP1-dependent) mitochondrial fission and a myeloid-biased, granulocyte-macrophage CSF–producing (GM-CSF–producing) progenitor state. The resulting neutrophil progeny further reinforced oxidative stress, thereby sustaining immunosuppressive myelopoiesis in the spleen. Disruption of this circuit through genetic, pharmacologic, or spleen-directed interventions reduced suppressive myeloid cell production, enhanced cytotoxic T cell activity, and restrained tumor growth. These findings establish mature neutrophils as active regulators of pathological splenic hematopoiesis and identify the neutrophil/ROS/FOXO1/DRP1 axis as a potential therapeutic target in cancer.

## Introduction

Cancer is increasingly understood as a systemic disease that reshapes host immunity and hematopoiesis beyond the primary tumor. Tumor-derived inflammatory signals can mobilize hematopoietic stem and progenitor cells (HSPCs) from the bone marrow and redirect these cells to extramedullary sites, particularly the spleen. There, HSPCs undergo myeloid-biased differentiation and generate neutrophils, monocytes, and myeloid-derived suppressor cells (MDSCs) that suppress antitumor immunity and promote tumor progression in situ and in metastasis sites (1–4).

The spleen is therefore more than a reservoir of mature immune cells. In tumor-bearing hosts, it can become a specialized hematopoietic niche that actively produces immunosuppressive myeloid cells (1, 2, 5). Previous work showed that splenic HSPCs are functionally distinct from their bone marrow counterparts and are particularly committed to generating suppressive neutrophils and MDSCs (2, 6). Recruitment of HSPCs through the CCL2/CCR2 axis, followed by local education by the splenic stroma, promotes endogenous granulocyte-macrophage CSF (GM-CSF) production and myeloid differentiation (2). Inhibition of this process can improve the efficacy of immune checkpoint blockade, emphasizing the importance of splenic hematopoiesis in therapeutic resistance (1, 5, 7).

However, an important question has remained unresolved: Once tumor-induced splenic myelopoiesis is established, how is it maintained? Tumor-derived cytokines and chemokines may initiate the process, but a continuously active tumor signal may not be required to sustain a remodeled hematopoietic niche. This possibility is supported by observations that tumor-educated splenic progenitors can retain myeloid-biased and immunosuppressive properties after transfer into tumor-free conditions (2, 6). It also raises the possibility that mature myeloid cells generated within the spleen may feed back on progenitors and perpetuate pathological hematopoiesis.

In this issue of the *JCI*, Zhu and colleagues identified a neutrophil-HSPC feedback circuit that explains such a mechanism (8). They show that tumor-associated splenic neutrophils generated NOX2-dependent ROS, which activated a Forkhead box O1/ dynamin-related protein 1 (FOXO1/DRP1) mitochondrial fission program in neighboring HSPCs. This mitochondrial remodeling promoted GM-CSF expression and myeloid-biased differentiation, thereby generating additional ROS-producing neutrophils. This work reframes tumor-associated splenic myelopoiesis as a self-propagating circuit rather than a linear response directed solely by tumor-derived or stromal signals.

## Neutrophils establish an oxidative splenic niche

Zhu et al. first examined the distribution of oxidative stress across tissues in several tumor models (8). The spleens of tumor-bearing mice displayed strikingly elevated ROS and hydrogen peroxide levels, exceeding those detected in the tumors and in several other organs. Among splenic immune cell populations, Ly6G^+^ neutrophils produced the highest levels of ROS, and neutrophil depletion markedly reduced splenic hydrogen peroxide concentrations.

These findings were complemented by spatial analyses showing that neutrophils and HSPCs were in closer proximity in tumor-bearing spleens than in control spleens. HSPCs located near neutrophils exhibited increased 4-hydroxynonenal, consistent with local oxidative damage. The result suggests that tumor-associated neutrophils were not simply accumulating in the spleen; rather, they were establishing an oxidatively stressed microenvironment in direct proximity to hematopoietic progenitors.

This observation is conceptually important because ROS have traditionally been considered intracellular mediators of damage or inflammatory signaling. In the present context, neutrophil-derived ROS function as a local intercellular signal that alters the fate of neighboring HSPCs. The spleen consequently becomes a site where mature myeloid cells can directly instruct the production of their own progeny.

## Mitochondrial fission reprograms HSPC fate

The authors next linked the oxidative splenic niche to HSPC function (8). Single-cell transcriptomics analyses revealed that splenic HSPCs from tumor-bearing mice were depleted of multipotent hematopoietic stem cell (HSC) states and enriched for a myeloid-biased early progenitor cell population. These cells displayed ROS response and inflammatory gene programs, together with mitochondrial stress and reduced oxidative phosphorylation. Spleen-directed ROS scavenging partially reversed these transcriptional changes.

At the structural level, splenic HSPCs from tumor-bearing animals exhibited fragmented mitochondria, reduced mitochondrial mass and membrane potential, increased mitochondrial superoxide, and decreased oxidative phosphorylation. This phenotype was largely restricted to the tumor-conditioned splenic niche, as bone marrow HSPCs from the same animals retained relatively intact mitochondrial networks.

Mechanistically, the authors identified DRP1-dependent mitochondrial fission as a central event. Neutrophils from tumor-bearing mice induced mitochondrial fragmentation in HSPCs ex vivo, whereas neutrophils lacking the NADPH oxidase 2 (NOX2) component encoded by *Cybb* did not. ROS scavengers similarly preserved mitochondrial integrity. These experiments support the idea of a causal pathway in which neutrophil-derived NOX2 activity induces mitochondrial remodeling in HSPCs.

FOXO1 provides an additional mechanistic link between oxidative stress and mitochondrial dynamics. In tumor-associated splenic HSPCs, FOXO1 accumulated in the nucleus in a ROS- and JNK-dependent manner. Pharmacologic or genetic inhibition of FOXO1 prevented DRP1 upregulation and mitochondrial fission. These observations establish a neutrophil ROS/JNK/FOXO1/DRP1 pathway that converts an extracellular oxidative signal into an intrinsic progenitor cell program.

The functional consequence of mitochondrial fragmentation was a shift toward immunosuppressive myelopoiesis. HSPCs with fragmented mitochondria expressed more GM-CSF, generated more granulocyte-macrophage colonies, and produced myeloid progeny with stronger T cell–suppressive activity. Inhibition of mitochondrial fission reduced GM-CSF expression, normalized colony output, and attenuated the suppressive function of the resulting myeloid cells. The authors further identified activation of the stimulator or IFN genes/TANK-binding kinase 1/NF-κB (STING/TBK1/NF-κB) pathway as a potential bridge between mitochondrial stress and *Csf2* transcription, although this mechanism requires additional validation.

## A feed-forward circuit sustains immune suppression

The central conceptual advance of the study is the demonstration that neutrophils and HSPCs form a positive-feedback loop by reprogramming HSPCs toward immunosuppressive, myeloid-biased differentiation (Figure 1). The adoptive transfer experiments conducted by Zhu et al. supported this model: Tumor-conditioned neutrophils promoted myeloid-biased output from otherwise naive HSPCs and established a splenic environment that remained instructive for newly introduced progenitors (8).

This finding extends previous models of splenic myelopoiesis. Earlier studies emphasized tumor-derived factors, HSPC recruitment, stromal signals, and progenitor-intrinsic pathways such as GM-CSF and PERK/ATF4/C/EBPβ signaling (2, 6, 9, 10). Zhu and colleagues add a further layer: The products of splenic myelopoiesis are not merely terminal effectors that leave the spleen; they actively maintain the upstream progenitor program.

The distinction between acute emergency hematopoiesis and chronic tumor-associated myelopoiesis is particularly relevant. In acute infection or tissue injury, feedback from mature myeloid cells may support a transient increase in granulopoiesis that resolves after the inciting stimulus is removed (11–13). In cancer, persistent tumor conditioning, oxidative stress, and altered splenic architecture may convert this adaptive response into a chronic, self-reinforcing program that preferentially produces suppressive myeloid cells (14).

## Therapeutic implications and remaining questions

Zhu and colleagues’ therapeutic experiments provide an important proof of principle (8). Spleen-directed ROS scavenging (*N*-acetyl-cysteine [NAC]) or inhibition of mitochondrial fission (Mdivi-1) reduced splenic HSPCs, GM-CSF–producing progenitors, and immunosuppressive neutrophils. These interventions increased IFN-γ–producing CD8^+^ T cells and slowed tumor growth, whereas delivery of the mitochondrial fission inhibitor to the tumor was less effective. Thus, targeting the hematopoietic source of suppressive cells may be more effective than targeting their downstream accumulation within the tumor.

However, several issues will need to be addressed before this strategy can be translated clinically. First, the specificity of ROS scavenging requires careful consideration. ROS are important for antimicrobial defense and physiological signaling, and systemic inhibition could compromise host protection (15, 16). Spleen-directed delivery, as used in this study, may provide a way to achieve greater selectivity, although clinically practical delivery platforms remain to be developed.

Second, pharmacologic tools such as Mdivi-1 should be interpreted cautiously because their specificity for DRP1 and mitochondrial fission has been debated (17, 18). Genetic perturbation of *Dnm1l*, HSPC-specific FOXO1 deletion, or more selective next-generation inhibitors will be important for confirming therapeutic tractability.

Third, the human data Zhu et al. presented are encouraging but preliminary. Fragmented mitochondria were observed in splenic HSPCs from only two patients with cancer, while the noncancer control group was small and clinically heterogeneous (8). Cord blood HSPC-neutrophil coculture experiments supported conservation of the ROS-responsive FOXO1/DRP1 pathway, but they did not fully reproduce the adult tumor-conditioned splenic niche. Larger studies should examine whether mitochondrial morphology, FOXO1 activation, DRP1 expression, or ROS-related transcriptional signatures in circulating or splenic progenitors correlate with disease stage, response to immunotherapy, or survival.

Finally, the identity of the neutrophil population responsible for initiating and maintaining the circuit needs to be addressed in future studies. Are these cells conventional neutrophils that acquire suppressive function in the spleen, polymorphonuclear neutrophil (PMN)-MDSCs, or a distinct progenitor-derived population? It will also be important to determine whether similar feedback loops operate in other extramedullary sites, including the liver, lungs, lymph nodes, or the tumor itself.

## Summary

Zhu and colleagues identify a self-sustaining neutrophil-HSPC circuit that maintains tumor-promoting splenic myelopoiesis. The study advances the field in 2 important ways. First, it establishes mature neutrophils as active regulators of pathological extramedullary hematopoiesis. Second, it identifies the mitochondrial state as a potentially targetable determinant of HSPC fate. The spleen thus emerges not only as a source of suppressive myeloid cells but also as a dynamic, self-reinforcing regulator of systemic antitumor immunity.

Future therapeutic strategies should aim to interrupt this circuit selectively — by targeting neutrophil-derived ROS/FOXO1/DRP1 signaling, or the communication between neutrophils and progenitors — while preserving normal host defense and hematopoiesis. More broadly, the findings suggest that overcoming resistance to immunotherapy may require targeting the hematopoietic supply line that continually replenishes the suppressive tumor microenvironment.

## Manuscript information

### AI disclosure.

The author declares that Claude Opus 5 was used for grammar checking and error corrections. The original figure was draw with BioRender.

### Conflict of interest.

HJ is co-author (McCray PB, Tack B, Jia HP, Schutte BC) of a patent titled “Human beta-defensin-3 (HBD-3), a highly cationic beta defensin antimicrobial peptide” (US patent no. 6809181, awarded 10/26/04); co-author (McCray PB, Davidson BL, Fischer AJ, Jia HP, Donovan MD, Sinn PL and Behlke MA) of a patent titled “RNA interference in respiratory epithelial cells” (US patent no. 7297786B2, awarded 11/20/07); co-author (Anderson, C, Jia, HP, Cui, HG) of a patent titled “Technology consists of inhalable supramolecular structures capable of docking and presentation of ACE2 enzyme on their surface, through which SARS coronavirus can be captured to prevent and treat infection” (Johns Hopkins University [JHU] Invention Disclosure, Invention ID: D16296, Tech ID: C16296; approved); co-author (Wang, C, Jia HP, Goyal MM) of a patent titled “Aerosol biosensors and related aspects for pathogen detection” (JHU application no. 63/026,594; approved); co-author (Guan, Yang, Jia HP, Lin, Liu) of a patent titled “Human HC1-his and HA-HC1-his: large-scale biosynthesis and applications” (JHU reference no. C19214).

### Funding.

This work was funded by the following:

- NIH grants R01AI199482, R01HL183181, and R01AI148446.

This work is the result of NIH funding, in whole or in part, and is subject to the NIH Public Access Policy. Through acceptance of this federal funding, the NIH has been given a right to make the work publicly available in PubMed Central.

## Figures and Tables

**Figure 1:**
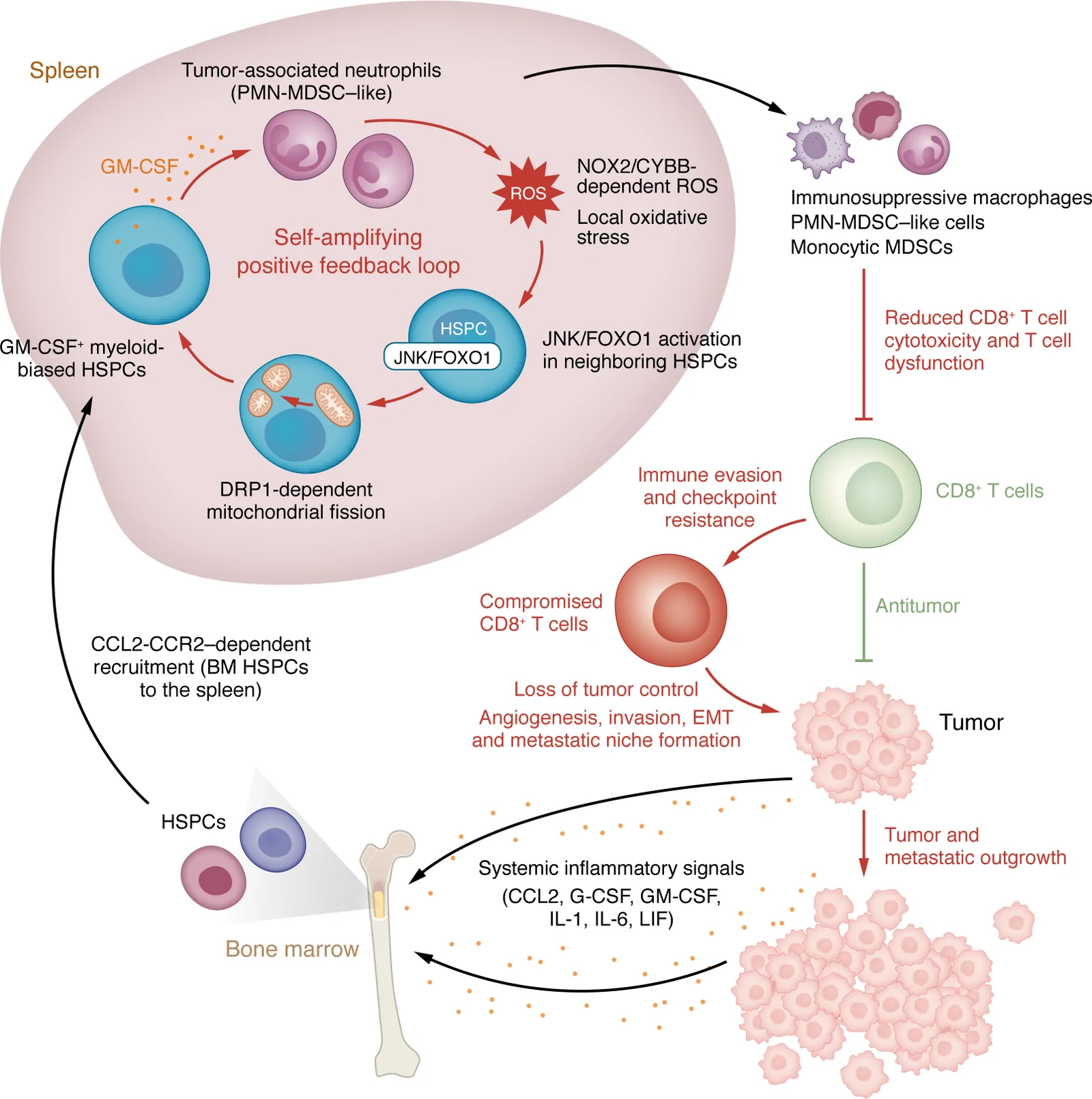
Splenic neutrophils sustain tumor-promoting extramedullary hematopoiesis. Zhu et al. (11) identified a feed-forward loop in which tumor-derived inflammatory signals mobilize bone marrow HSPCs and recruit them to the spleen, where neutrophil-derived, NOX2-dependent ROS activate JNK/FOXO1- and DRP1-dependent mitochondrial fission in neighboring progenitors. The resulting GM-CSF–producing, myeloid-biased HSPCs generate further immunosuppressive neutrophils, MDSCs, and macrophages, establishing a self-amplifying circuit that compromises CD8^+^ T cell cytotoxicity and supports immune evasion, angiogenesis, and metastasis. EMT, epithelial-mesenchymal transition; LIF, leukemia inhibitory factor.

## Version 1 11/16/2026

Electronic publication
